# Poor Social Functioning: A Potentially Modifiable Risk Factor for Pneumonia in the Elderly

**DOI:** 10.7759/cureus.47520

**Published:** 2023-10-23

**Authors:** Sugihiro Hamaguchi, Sho Sasaki, Sayaka Shimizu, Hajime Yamazaki, Ryohei Yamamoto, Akihiro Ozaka, Hiroaki Nakagawa, Taro Takeshima, Joseph Green, Shunichi Fukuhara

**Affiliations:** 1 Department of General Internal Medicine, Fukushima Medical University, Fukushima, JPN; 2 Center for Innovative Research for Communities and Clinical Excellence (CiRC2LE), Fukushima Medical University, Fukushima, JPN; 3 Section of Clinical Epidemiology, Department of Community Medicine, Graduate School of Medicine, Kyoto University, Kyoto, JPN; 4 Research Section, Patient Driven Academic League (PeDAL), Tokyo, JPN; 5 Department of General Medicine, Shirakawa Satellite for Teaching and Research (STAR), Fukushima Medical University, Shirakawa, JPN; 6 Center for University-Wide Education, School of Health and Social Services, Saitama Prefectural University, Saitama, JPN; 7 Graduate School of Medicine, The University of Tokyo, Tokyo, JPN; 8 Department of Health Policy and Management, Johns Hopkins Bloomberg School of Public Health (JHSPH), Baltimore, USA

**Keywords:** community-acquired pneumonia, risk factors for pneumonia, pneumonia in the elderly, social functioning, health-related quality of life

## Abstract

Background

Most risk factors for developing community-acquired pneumonia (CAP) are age-related and chronic medical conditions; modifying these factors can be challenging, especially in the elderly. Poor social functioning, however, has a negative impact on medical conditions but can be improved through interventions. Therefore, the social functioning domain of health-related quality of life (HRQOL) may be a modifiable risk factor for the development of CAP. This study investigated the association between poor social functioning and the incidence of CAP in elderly individuals.

Methodology

We conducted a retrospective cohort study using a dataset from 2018 to 2021, derived from an annual questionnaire-based survey of a cohort of community-dwelling people aged 75 years or older (the Sukagawa Study). The dataset included social functioning subscale scores of HRQOL obtained from the Eight-Item Short Form (SF-8) questionnaire. Health insurance claims data were matched with these HRQOL data. For each participant, the exposure (HRQOL) was measured, and outcomes (incidence of CAP) were observed yearly from 2018 through 2021.

Results

The four observation years had a total of 17,016 observation periods among 6,513 participants. The annual incidence rate of CAP was 0.90-1.77%. Lower social functioning was associated with a higher risk of CAP. Specifically, for each standard deviation difference in social functioning score, the adjusted rate ratio for CAP incidence was 1.26 (95% confidence interval (CI) = 1.08-1.48). In a subgroup analysis, the association between social functioning and CAP differed by sex (p = 0.037). Specifically, the adjusted rate ratio for CAP incidence was 1.41 (95% CI = 1.17-1.70) in men and 1.00 (95% CI = 0.76-1.35) in women.

Conclusions

Poor social functioning is an important risk factor for CAP in the elderly, especially in men. Interventions that improve social functioning may help to prevent CAP.

## Introduction

In Japan, pneumonia is an important cause of morbidity and mortality. In part because of Japan’s current demographic structure and trends, most risk factors for death among patients in Japan who have community-acquired pneumonia (CAP) are age-related, chronic conditions [1]. The result is that preventing patients with CAP from dying is often difficult [2]. Therefore, in a society with a large proportion of elderly people, it might be more effective to put some emphasis on preventing pneumonia rather than focusing only on preventing death after pneumonia is diagnosed.

Risk factors for CAP include age, smoking, environmental exposure (e.g., metal, dust, fumes), malnutrition, previous CAP infection, chronic obstructive pulmonary disease (COPD)/asthma, functional impairment, poor dental health, use of immunosuppressive therapy, and use of gastric-acid-suppressing drugs [3]. However, modifying those risk factors is either impossible (e.g., age) or challenging, especially in elderly individuals. To reduce the incidence of CAP in rapidly aging countries such as Japan, additional studies are needed to identify modifiable risk factors.

Pneumonia is associated with reduced health-related quality of life (HRQOL) in elderly patients [4-6]. In most research on this topic, HRQOL is the outcome variable, but poor HRQOL can be a causal risk factor for some medical conditions. For example, poor HRQOL was found to be a predictor of hospitalization and mortality among patients undergoing hemodialysis [7], while a separate study showed that having more social ties was associated with greater resistance to upper respiratory tract infections [8]. In addition, the results of a pilot randomized controlled trial conducted during the coronavirus disease 2019 (COVID-19) pandemic demonstrated that in older adults an eight-week online interactive course using LINE (a freeware app for instant communications with exchanging texts, images, videos, and audios on electronic devices) reduced loneliness, as well as improved scores on the HRQOL domains of psychological health and social relationships [9]. Together, these findings suggest that the social aspect of HRQOL is modifiable and that it may be a risk factor for CAP.

Additionally, some studies have identified that being male is a risk factor for both pneumonia incidence and mortality, albeit for reasons that remain unknown [2,10]. In light of sex disparities observed in social participation [11], it is postulated that sex-specific variations may exist in the association between the social aspect of HRQOL and the incidence of pneumonia.

In this retrospective cohort study, we examined the relationship between the social aspect of HRQOL and the incidence of CAP in elderly individuals. We also conducted a subgroup analysis defined by sex to investigate sex-specific differences.

## Materials and methods

Participants and setting

Much of the data came from the Sukagawa Study, a community-based cohort study that includes individuals aged 75 years or older residing in Sukagawa City, Fukushima Prefecture, Japan. According to the cohort profile of the Sukagawa Study, Sukagwa city is considered a representative of the provincial cities in Japan in terms of research purposes [12]. The data were collected over four years, i.e., 2018 to 2022. To address gaps in the data, we also incorporated information obtained in the same study in 2015, 2016, and 2017 using multiple imputations. The Sukagawa Study is an annual questionnaire-based survey of all Sukagawa City residents aged 75 years or older who voluntarily provide consent to participate during the initial questionnaire administration. Data were collected using paper-based instruments. Comprehensive social and health-related data were collected.

After consenting to enrollment in the registry, participants received documents outlining their prerogative to withdraw consent at any time, along with a consent withdrawal form. Data derived from the questionnaires were later combined with demographic data; disease-related information according to the International Classification of Diseases, Tenth Revision (ICD-10); prescription drug records; details regarding nursing care; and periodic updates on mortality and relocation status sourced from the data of the Healthcare Claims Database, healthcare institutions, and government authorities.

This cohort has already been described in detail [12]. Data regarding individuals who had dementia and those who needed at least a moderately high level of nursing care (level 3 or higher, see below) were excluded from the analysis because their social functioning was already minimal. The diagnosis of dementia was based on the Mini-Cog [13], a dementia screening instrument administered yearly, with a score of 2 or less indicating the presence of dementia. The nursing care levels used were those of Japan’s long-term care insurance system [14] which is under the purview of municipal administrations and classifies nursing care needs into five levels, with level 1 indicating the least disabled and level 5 indicating the most disabled.

Exposure

The exposure was the score on the Social Functioning question item of the Eight-Item Short Form (SF-8) [15]. The SF-8 is a self-administered questionnaire that has one item to measure each of the eight domains of HRQOL, namely, physical functioning, role-physical, bodily pain, general health, vitality, social functioning, role-emotional, and mental health. It has been studied psychometrically and clinically and has been used in Japan [16]. SF-8 data were scored using standardized national norms. The scores can be interpreted by comparison with scores from a representative sample of the general population of Japan, with mean = 50, and one standard deviation (SD) = 10.

Outcome

The outcome was the incidence rate of CAP during each year-long observation period. CAP was defined as either a simple chest X-ray image or computed tomography (CT) scan obtained during an outpatient visit including that with subsequent emergency hospitalization on the same day, which was consistent with one of the following ICD-10 diagnoses: lobar pneumonia, diffuse pneumonia, acute pneumonia, senile pneumonia, bacterial bronchopneumonia, influenza rod pneumonia, pneumococcal pneumonia, rod pneumonia, staphylococcal pneumonia, methicillin-resistant *Staphylococcus aureus* pneumonia, enterococcal pneumonia, *Pseudomonas aeruginosa* pneumonia, mycoplasma pneumonia, legionella pneumonia, and COVID-19 pneumonia.

Study design

**Figure 1:**
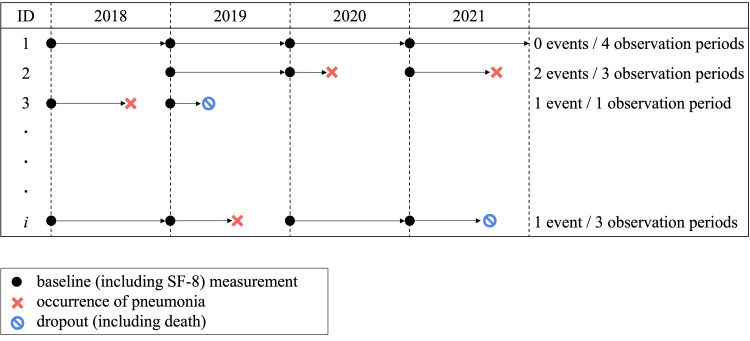
Study design. Data were collected over four observation periods, each of which lasted for one year. Eight-Item Short Form (SF-8) data were collected from questionnaires distributed in March of each year. Each one-year period of observation for incident cases of community-acquired pneumonia began on April 1.

The exposure and the outcome were measured once each year, as shown in Figure 1. SF-8 data were collected from questionnaires distributed in March of each year. Each one-year period of observation for incident cases of CAP began on April 1.

Confounders

Based on previous research, especially based on evidence from the systematic review [3], we considered the following 12 variables to be potential confounding factors: age, sex, current smoking status, malnutrition, history of pneumonia, COPD, asthma, peptic ulcer, care level (an equivalent of functional impairment) [14], the number of remaining teeth (as a proxy for general oral hygiene), rheumatic disease, and malignancy. All of these variables were assessed at the time of exposure measurement. Malnutrition was defined as a body mass index of less than 18.5 kg/m^2^, according to the criteria of the European Society of Clinical Nutrition and Metabolism [17]. Pneumonia, COPD, asthma, peptic ulcer, rheumatic disease, and malignancy were considered present if their ICD-10 codes could be confirmed using insurance claim data. The number of remaining teeth was classified as 0, 1-9, 10-19, or 20 or more. Peptic ulcer was considered to be present if the participant had received a prescription for a proton pump inhibitor, an H2 antagonist, or a potassium-competitive acid blocker.

Ethics approval

This study adhered to the tenets of the Declaration of Helsinki and was approved by the Internal Ethics Review Board of Fukushima Medical University (approval number: 2975). The ethics board waived the requirement for written informed consent because this study was retrospective.

Statistical analysis

Descriptive analysis was used to describe the patients’ backgrounds at the beginning of the observation periods. Normally distributed continuous variables are described as mean and SD or as median and interquartile range. Non-normally distributed and categorical variables are described as the number and proportion.

To verify the association between exposure and outcomes, we estimated the rate ratio using generalized estimating equations (GEEs) with robust analysis of variance. This approach accounted for the clustering of data within individuals. The rate ratio was estimated using the log link function and the Poisson distribution model. First, the crude relationship between the exposure and outcome was estimated, followed by multivariate analysis. The multivariate model included adjustments for the confounding factors described above. Because dropouts, including deaths, competed with the outcome, the observation period during which a dropout occurred and subsequent information were excluded from the analysis (Figure 1).

We also analyzed data in sex-defined subgroups. In this analysis, we used a model in which sex was excluded from the explanatory variables. We also obtained p-values for the interaction between sex and social functioning subscale scores from that model.

Missing data, except for missing exposure and outcome data, were handled with multiple imputation by chained equation (MICE) [18]. To increase the precision of estimates from that process, MICE also used Sukagawa Study data obtained in 2015, 2016, and 2017, which were not used in other analyses. Two-tailed p-values of less than 0.05 were taken to indicate statistical significance. All statistical analyses were done using Stata version 18.0 (Stata Corp., College Station, TX, USA).

## Results

Study flow and baseline characteristics

**Figure 2:**
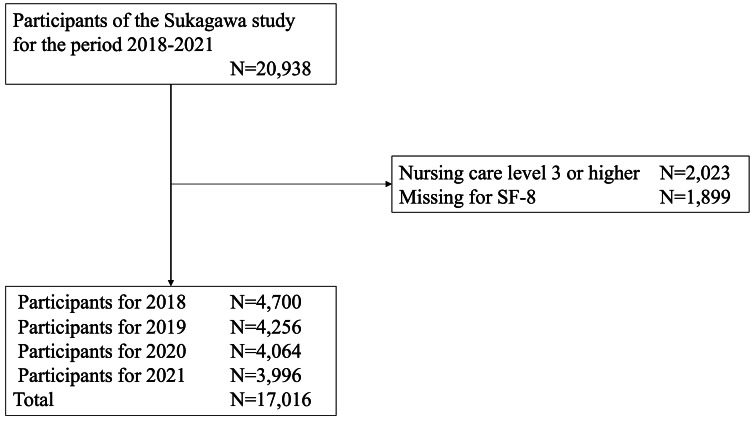
Study flowchart.

As shown in Figure 2, 17,016 (n = 6,513) of the 20,938 (n = 7,480) observation periods in the Sukagawa Study from 2018 to 2021 were included in the analysis. The baseline characteristics of the participants are summarized in Table 1. The annual incidence of CAP was 0.90% to 1.77% from 2018 to 2021.

**Table 1:** Baseline characteristics. SD: standard deviation; CAP: community-acquired pneumonia; COPD: chronic obstructive pulmonary disease

	Measured	Missing
	n = 17,016	
Male	7,646 (44.9%)	0
Age in years (mean and SD)	81.0 (4.6)	0
Body mass index (mean and SD)	23.3 (3.2)	3,034 (17.8%)
Smoking status		585 (3.4%)
Non-smoker	10,067 (59.2%)	
Past smoker	5,294 (31.1%)	
Current smoker	1,070 (6.3%)	
Teeth remaining		12,457 (73.2%)
0	935 (5.5%)	
1–9	1,193 (7.0%)	
10–19	1,079 (6.4%)	
20+	1,352 (7.9%)	
Nursing care level		28 (0.2%)
0	16,899 (99.3%)	
1	51 (0.3%)	
2	38 (0.2%)	
Known risk factors for CAP		
History of pneumonia	658 (3.9%)	0
Asthma	4,244 (25.2%)	0
COPD	6,073 (35.7%)	0
Rheumatic disease	953 (5.6%)	0
Peptic ulcer disease	3,148 (18.5%)	0
Cancer	3,658 (21.5%)	0
Metastatic cancer	569 (3.3%)	0

Number of cases of CAP

In 2018, 2019, 2020, and 2021, the annual incidence rates of CAP were 1.77, 1.55, 0.94, and 0.90, respectively. During the four years of this study, 223 cases of CAP were recorded: each of the 195 participants had one case and each of the 14 participants had two cases.

Association between social functioning score and CAP incidence

According to the results of the univariate analysis, having social functioning scores lower than the national norm was directly related to CAP incidence. That relationship was robust to the adjustments for confounders included in the multivariate model (Table 2).

**Table 2:** Association between social functioning score and incidence of CAP in the subsequent year. In multivariate analysis, we adjusted for age, sex, current smoking status, malnutrition, history of pneumonia, COPD, asthma, peptic ulcer, level of care, number of remaining teeth, rheumatic disease, and malignancy. The point estimates are rate ratios of CAP incidence per one-SD-lower-than-the-national-norm score on the SF-8 social functioning item. CAP: community-acquired pneumonia; CI: confidence interval; COPD: chronic obstructive pulmonary disease; SD: standard deviation; SF-8: Eight-Item Short Form

	Point estimate	95% CI
Unadjusted rate ratio	1.45	1.23–1.70
Multivariate-adjusted rate ratio	1.26	1.08–1.48

Subgroup analysis by sex

According to the results of the subgroup multivariate analysis, the association between low social functioning scores and subsequent CAP incidence was stronger among men than among women. The interaction between social functioning scores and sex was statistically significant (p = 0.037) (Table 3).

**Table 3:** Association between social functioning score and CAP incidence, stratified by sex. In multivariate analysis, we adjusted for age, current smoking status, malnutrition, history of pneumonia, COPD, asthma, peptic ulcer, level of care, number of remaining teeth, rheumatic disease, and malignancy. The point estimates are multivariate-adjusted rate ratios of CAP incidence per one-SD-lower-than-the-national-norm score on the SF-8 social functioning item. The interaction between social functioning scores and sex was statistically significant (p = 0.037). CAP: community-acquired pneumonia; CI: confidence interval; COPD: chronic obstructive pulmonary disease; SD: standard deviation; SF-8: Eight-Item Short Form

Multivariate-adjusted rate ratio	Point estimate	95% CI
Men	1.42	1.17–1.70
Women	1.01	0.76–1.35

## Discussion

The results presented above show that poor social functioning was associated with the subsequent incidence of CAP. Moreover, the association between social functioning and CAP was stronger in men than in women. Both of these results came from analyses that were adjusted for age and other likely confounders.

In the participants as a whole, having a social functioning score that was one SD (i.e., 10 points) lower than the Japanese national norm was associated with a 26% higher risk of CAP incidence in the following year. The subgroup analysis showed that the higher risk of CAP incidence mentioned above was confined almost completely to men. Specifically, low social functioning in men was associated with a 41% higher risk of CAP incidence in the following year, whereas in women the size of that effect was almost zero.

To our knowledge, no previous study has assessed HRQOL as a potential risk factor for CAP in elderly people. We found only one study in which QOL was used as a potential risk factor for CAP development [19]. However, in that study the performance status (PS) index [20] was used as a surrogate for QOL, and the CI for the adjusted odds ratio included 1: 3.17, 95% CI = 0.55-18.04. The PS index reflects physical impairment in cancer patients; it is not a patient-reported outcome measure and is instead typically assessed by healthcare providers. It is also unidimensional [20]. Therefore, the PS index is not considered to be useful as a substitute for a measure of HRQOL. In contrast, the SF-8 is a patient-reported and multidimensional measure that includes an item asking directly about the social aspect of HRQOL. Furthermore, physical functional impairment, which is the main component of the PS index, is a known risk factor for CAP [3]. In contrast, we focused on the social aspect of HRQOL.

Social functioning can be greatly influenced by the quality and quantity of social connections and relationships [21]. A meta-analysis of 148 studies found an association between social relationships and mortality [22]. Considering that pneumonia is among the leading causes of death in elderly people, it is plausible that social functioning is linked to pneumonia-related mortality.

As noted above, the association between poor social functioning and subsequent CAP incidence was much stronger in men than in women. Some studies have identified that women have more social interaction related to health than men and that women may benefit from social capital more than men [23,24]. Therefore, the minimal association with the occurrence of pneumonia in women may be explained by the potential protective effect of stronger social relationships. A recent study classified elderly people in Japan into one of the following three groups: “Active,” “Socially isolated,” or “Less motivated,” and found that men were more likely to be in the “Socially isolated” group. People in that group had the lowest probability of interacting with others, limited social participation, and a reduced sense of safety [25]. Thus, older men in Japan have relatively poor social relationships, which appears to put them at a greater risk of CAP incidence.

The results of this study have at least one important practical implication: Interventions aimed at improving social functioning might prevent some cases of CAP. Social functioning is a multidimensional domain of HRQOL which is strongly affected by social relationships. Therefore, it can potentially be improved through some interventions to increase social interaction. For example, a pilot study of a randomized controlled trial showed that an eight-week online interactive course using LINE reduced loneliness and improved the psychological health and social relationship domains of QOL among elderly individuals during the COVID-19 pandemic [9]. Further, a community-based interventional longitudinal study found that more frequent participation in eight-week social interaction sessions improved the QOL of elderly individuals when compared with less frequent participation. Therefore, interventions designed to improve the social functioning of elderly individuals, particularly men with poor social functioning, may help to prevent CAP.

Our study has some limitations. First, for outcome measurement (CAP incidence), we used the data derived from insurance claim data. Therefore, in some cases, the recorded diagnosis may differ from the actual medical condition. However, the outcome criterion included the requirement for chest radiography, which should reduce the likelihood of false positives and false negatives. Furthermore, the use of ICD-10 codes to notate medical records of pneumonia has been reported to be a valid method and superior to the use of symptoms and signs or radiological findings [26]. Therefore, we believe that the outcome definition we used in our study was not out of standard for research purposes. Second, the potential confounding factors of immunosuppressant use and antacid use were replaced with proxies (rheumatic disease, cancers, and peptic ulcer disease) owing to the absence of prescription information. Given that those disorders are very frequently accompanied by the use of the drugs in question, we believe that using those proxies is unlikely to have introduced important bias. Third, we used SF-8, an abbreviated version of the SF-36. Although the SF-36 is a well-established, reliable, and valid HRQOL instrument that is used worldwide, including 36 more items in the already very limited space of the questionnaire for this community-based survey was challenging. We found the SF-8 to be much more feasible in this setting. Importantly, when using the SF-8 the compromise is minimal. Not only are the SF-36 and SF-8 in the same lineage but both have comparable reliability and validity [27]. Fourth, among confounders, the remaining teeth had several missing data (73.2% missing). These missing measurements were due to the fact that the number of remaining teeth was measured only in 2018. In other words, the number of remaining teeth was an item that was not missing for 97% of the 4,700 subjects in 2018. As oral health is a significant risk factor for developing pneumonia according to the systematic review, MICE was used to address the missing number of remaining teeth to minimize bias in the results [28]. Finally, environmental factors (e.g., dust exposure) are known to be risk factors for CAP incidence but were not measured. Previous studies have utilized regional values, such as particulate matter 2.5, rather than patient-specific information, for these factors. As we used data from a questionnaire survey and health insurance claims, measurement of these factors was not feasible.

## Conclusions

Poor social functioning may be a modifiable risk factor for CAP incidence in elderly people, particularly in men. In these people, the hypothesis that interventions to improve social functioning will reduce the incidence rate of CAP should be tested.
